# Treatment decisions and mortality in HIV-positive presumptive smear-negative TB in the Xpert® MTB/RIF era: a cohort study

**DOI:** 10.1186/s12879-017-2534-2

**Published:** 2017-06-16

**Authors:** Sabine M. Hermans, Juliet A. Babirye, Olive Mbabazi, Francis Kakooza, Robert Colebunders, Barbara Castelnuovo, Christine Sekaggya-Wiltshire, Rosalind Parkes-Ratanshi, Yukari C. Manabe

**Affiliations:** 1 Infectious Diseases Institute, Makerere University College of Health Sciences, P.O. Box 22418, Kampala, Uganda; 2 Department of Internal Medicine, School of Medicine, Makerere University College of Health Sciences, Kampala, Uganda; 3 Department of Global Health, Academic Medical Center, University of Amsterdam, Amsterdam Institute for Global Health and Development, Amsterdam, The Netherlands; 4 Institute for Tropical Medicine, University of Antwerp, Antwerp, Belgium; 5 Global Health Institute, University of Antwerp, Antwerp, Belgium; 6 Institute of Public Health, University of Cambridge, Cambridge, UK; 7 Division of Infectious Diseases, Department of Medicine, Johns Hopkins University School of Medicine, Baltimore, USA

**Keywords:** Empirical treatment, Molecular diagnostic techniques/methods, Tuberculosis, pulmonary/diagnosis, Tuberculosis, pulmonary/epidemiology, HIV Infections/complications

## Abstract

**Background:**

The Xpert® MTB/RIF (XP) has a higher sensitivity than sputum smear microscopy (70% versus 35%) for TB diagnosis and has been endorsed by the WHO for TB high burden countries to increase case finding among HIV co-infected presumptive TB patients. Its impact on the diagnosis of smear-negative TB in a routine care setting is unclear. We determined the change in diagnosis, treatment and mortality of smear-negative presumptive TB with routine use of Xpert MTB/RIF (XP).

**Methods:**

Prospective cohort study of HIV-positive smear-negative presumptive TB patients during a 12-month period after XP implementation in a well-staffed and trained integrated TB/HIV clinic in Kampala, Uganda. Prior to testing clinicians were asked to decide whether they would treat empirically prior to Xpert result; actual treatment was decided upon receipt of the XP result. We compared empirical and XP-informed treatment decisions and all-cause mortality in the first year.

**Results:**

Of 411 smear-negative presumptive TB patients, 175 (43%) received an XP; their baseline characteristics did not differ. XP positivity was similar in patients with a pre-XP empirical diagnosis and those without (9/29 [17%] versus 14/142 [10%], *P* = 0.23). Despite XP testing high levels of empirical treatment prevailed (18%), although XP results did change who ultimately was treated for TB. When adjusted for CD4 count, empirical treatment was not associated with higher mortality compared to no or microbiologically confirmed treatment.

**Conclusions:**

XP usage was lower than expected. The lower sensitivity of XP in smear-negative HIV-positive patients led experienced clinicians to use XP as a “rule-in” rather than “rule-out” test, with the majority of patients still treated empirically.

## Background

Tuberculosis (TB) remains the main cause of death among HIV-infected patients in sub-Saharan Africa. Sputum smear microscopy has a low sensitivity for detecting TB particularly among people living with HIV. Because of its low cost, it is the mainstay of diagnosis in many high burden countries. In December 2010 the World Health Organization (WHO) endorsed Xpert® MTB/RIF (Cepheid, Sunnyvale, California, USA) (XP) [1], the first fully automated, real-time nucleic acid amplification technology for the rapid detection of TB [1, 2] which can be performed with only 1 day of training by most health care workers. A WHO policy statement came out in 2011 recommending its use as the initial diagnostic test among people suspected of multi-drug resistant (MDR) or HIV-associated TB [2]. WHO policy updates in October 2013 recommending broad use of Xpert for TB diagnosis [3] and concessionary pricing for high-burden countries have resulted in widespread roll-out in routine care settings [4].

Because of a higher sensitivity than sputum smear microscopy and a running time of two hours, routine use of XP was predicted to increase case finding and improve TB treatment outcomes [5–7]. However, this impact has not been realized in trials to date [8–11]. Some have argued that the impact may been blunted by high rates of empirical treatment in sub-Saharan Africa: up to 40% of TB cases are treated empirically due to diagnostic uncertainties and the risk of severe morbidity and mortality if TB treatment is delayed [12]. It is therefore still unclear what impact XP will have on case finding and TB treatment outcomes in routine care settings [13]. To date no studies have investigated the impact of XP on health care workers’ pre- and post-test management [13].

In an integrated TB-HIV clinic with adequate staffing and training in Kampala, Uganda, we implemented XP for use as an add-on test in sputum smear negative presumptive TB patients. We investigated if and how clinicians changed their diagnostic decision-making on the basis of the additional test available to them. Considering the high level of experience of the clinicians, we hypothesized that this would be limited. We analyzed XP usage and yield compared to empirical diagnosis without access to XP, and examined treatment decisions subsequent to an XP result. We also compared mortality in the first year after being investigated for TB between patients who were treated empirically, treated on the basis of microbiological confirmation, or not treated at all.

## Methods

### Study setting

This study was performed at the integrated adult TB/HIV clinic of the Infectious Diseases Institute, part of the Makerere University College of Health Sciences located at Mulago Hospital in Kampala, Uganda. This clinic has been described in detail previously [14, 15]. In brief, this clinic provided a one-stop-shop outpatient TB and HIV service for all patients suspected of having TB among the over 30,000 registered HIV-positive patients of the Adult Infectious Diseases Clinic. It is staffed by medical officers, nurses and counsellors who provide care for both TB and HIV during the same clinic visit.

All patients were screened for TB symptoms in the overall HIV clinic waiting area, in accordance with guidelines of the WHO and the Ugandan Ministry of Health [15]. Those who screened positive were referred to the TB/HIV clinic, where the clinicians followed local guidelines to diagnose TB. At the time of the study, 2 sputum samples were tested with smear microscopy, one collected immediately and one on the following morning. Additional available investigations included chest X-ray, lymph node aspirate and abdominal ultrasound where applicable (fee-for-service). Sputum cultures were not routinely available. Treatment of smear-negative patients was based on the clinician’s interpretation of any ongoing symptoms and the chest X-ray. Prior to XP implementation, 22% of patients treated for TB were smear-negative [14, 15]. At the time of the study, although the green light committee of the WHO had approved Uganda for multi-drug resistance (MDR) TB treatment, drugs were not yet available. All patients in whom MDR was detected were referred to the national TB clinic in Mulago Hospital, to await the arrival of the drugs.

### Study design and population

This was a prospective cohort study in which we included all presumptive TB patients of the TB/HIV clinic who were sputum smear microscopy negative from 1 May 2012–1 May 2013. Clinicians could order an XP using a request form which included the following question: “with the current clinical and diagnostic information available to you, would you start this patient on TB treatment?”, which was to be answered by ticking a box either “yes” or “no”. Clinicians were trained how to use the form and there were ongoing checks of their understanding. The XP was performed immediately while the patient waited for the results. The result was returned to the clinician; the request forms were retained in the laboratory. Treatment was initiated on the same day according to the clinician’s interpretation of the XP result. Patients were followed up for 1 year to determine their outcome.

### Ethical review statement

The Adult Infectious Diseases Clinic has ongoing approval by the Makerere University Research Ethics Committee and the Uganda National Council of Science and Technology to utilize routinely collected data for operational research purposes. A written consent waiver to do retrospective analysis of routinely collected data was granted; data were analysed after removal of unique personal identifiers.

### Data management and definitions

The results on the XP laboratory request forms were entered into a study database by a laboratory technician and then merged with routinely collected clinical, diagnosis and treatment electronically collected data of the TB/HIV clinic. A *pre-XP empirical diagnosis* was defined as the intention to treat for TB before access to the XP result (at the time of XP request). *Post-XP empirical TB treatment* was defined as the decision by a clinician to treat for TB without a positive XP result. We further categorized patients according to their final diagnostic and treatment profile: TB treatment on the basis of microbiological confirmation (positive XP), post-XP empirical treatment (negative or no XP) or no treatment (negative or no XP).

During the period of our study the CD4+ T cell (CD4) counts were only determined once a year. We therefore used the closest measurement within 6 months before or after the date of presentation as a presumptive TB patient. HIV viral load monitoring was not routinely available.

### Statistical methods

We used descriptive statistics for the baseline characteristics of the study population at first presentation to the TB clinic, and to determine XP usage (the proportion of patients who were evaluated with XP) and to compare diagnostic and treatment decisions. Differences were tested using Chi-2, Fisher’s Exact, Student’s t-test, Kruskal-Wallis or Wilcoxon rank sum tests, as appropriate. Logistic regression was used to calculate the odds ratio of TB treatment by pre-XP empirical diagnosis, overall and stratified by XP result. To prevent over-fitting of the model due to the small number of outcomes in this group only multivariable analyses with two independent variables was done. Survival analysis, the log-rank test for equality of survivor functions and Cox regression were used to compare mortality in the first year after the presentation at the TB/HIV clinic by the different diagnostic and treatment profiles. The proportional hazards assumption was checked using log(−log(survival)) curves and Schoenfeld residuals. Adjusted hazard ratios (aHR) of the risk of death in the first year were calculated using Cox regression and adjusting for a priori risk factors (sex, CD4 count and ART at date of presumptive TB). Age was omitted due to risk of over-fitting; initial univariable analysis showed no association however. Data were analysed using STATA 12.0 SE (StataCorp, College Station, Texas, USA).

## Results

A total of 495 patients were evaluated for tuberculosis with a sputum smear between 1 May 2012 and 1 May 2013. Of these, 84 (17%) were sputum smear-positive. The 411 (83%) patients who were sputum smear-negative were included in this analysis. The median age of the study participants was 38 years (interquartile range 31, 44), 56% were female, 15% had been previously treated for TB, 50% were on ART at the time of investigation, and the median CD4 count at presentation was 275/mm^3^ (interquartile range, 141–440) (Table 1).

**Table 1:** Baseline characteristics of smear-negative presumptive TB patients, overall and by whether they had received an Xpert® MTB/RIF

Characteristic		No XP	XP	Total	*P*-value
Total (*n* [%])		236 (57.4)	175 (42.6)	411 (100)	
Age (mean [SD])		38 (9)	40 (11)	39 (10)	0.10
Female sex (*n* [%])		133 (56.4)	99 (56.6)	232 (56.4)	0.97
CD4 count (median [IQR])*		274 (147, 422)	275 (179, 478)	275 (162, 426)	0.73
ART at TB treatment initiation (*n* [%])		116 (49.2)	89 (50.9)	205 (49.9)	0.73
Prior TB treatment (*n* [%])		34 (14.4)	29 (16.6)	63 (15.3)	0.55
Symptomatology at presentation (*n* [%])	Cough	228 (96.6)	173 (98.9)	401 (97.6)	0.14
	Fever	169 (71.6)	124 (70.9)	293 (71.3)	0.87
	Night sweats	112 (47.5)	88 (50.3)	200 (48.7)	0.57
	Weight loss	80 (33.9)	63 (36)	143 (34.8)	0.66
	Anorexia	113 (47.9)	84 (48)	197 (47.9)	0.98
	Chest pain	64 (27.1)	57 (32.6)	121 (29.4)	0.23

*IQR* interquartile range, *n* number, *SD* standard deviation, *TB* tuberculosis, *XP* Xpert® MTB/RIF

*On 200, 152 and 352 patients, respectively

**Fig. 1:**
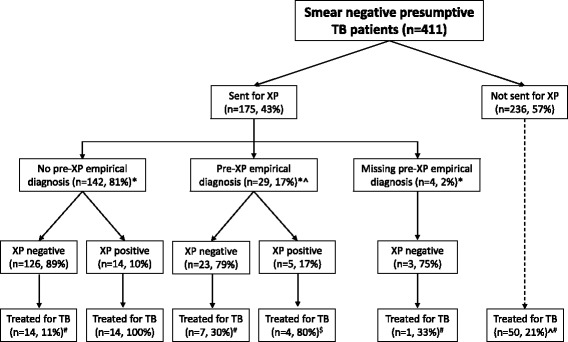
Flow diagram of study patients. A pre-XP empirical diagnosis was defined as the recorded intention to start treatment for TB without access to XP (as determined by the clinician at the time of the XP request). TB, tuberculosis; XP, Xpert® MTB/RIF. *Invalid XP results in 2, 1 and 1 patients respectively. ^Patients treated empirically pre-XP. ^#^Patients treated empirically post-XP. ^$^1 patient died before being started on TB treatment

A flow diagram outlining the diagnostic process and treatment decisions in the included patients is shown in Fig. 1. Among the 411 smear-negative presumptive TB patients, 175 (43%) were sent for an XP. There were no technical problems with the machine during the study period. There were no differences in baseline characteristics between patients who did or did not receive an XP test, including symptomatology at presentation (Table 1). However, patients who underwent an XP received a chest X-ray more often (94% versus 78%, *P* < 0.001) which was more likely to be abnormal (69% versus 54%, *P* 0.008). Patients who received an XP were equally likely to be diagnosed with TB (23% versus 21%, *P* 0.59) as those who did not, but were less likely to be diagnosed with extra-pulmonary TB (17% versus 50%, *P* 0.001).

The minority of the patients for whom an XP was requested received a pre-XP empirical diagnosis (29 [17%]). Nonetheless, there were no differences in baseline characteristics among patients with and without a pre-XP empirical diagnosis (Table 2). There was also no difference in XP positivity (17% versus 10%, *P* = 0.23). We identified rifampicin resistance in one of the patients without a pre-XP empirical diagnosis.

**Table 2:** Baseline characteristics of smear-negative presumptive TB patients who received an Xpert® MTB/RIF by whether the clinician would have treated them without access to the Xpert result or not

Characteristic		No pre-XP empirical diagnosis	Pre-XP empirical diagnosis	Missing response	Total	*P*-value
Total (*n* [%])		142	29	4	175	
Age (mean[SD])		40 (11)	39 (9)	47 (11)	40 (11)	0.52
Female sex (*n* [%])		80 (56.3)	16 (55.2)	3 (75)	99 (56.6)	0.75
CD4 count (median [IQR])*		280 (189, 483)	238 (132, 343)	403 (390, 983)	275 (179, 478)	0.66
ART at TB treatment initiation (*n* [%])		72 (50.7)	16 (55.2)	1 (25)	89 (50.9)	0.53
Prior TB treatment (*n* [%])		23 (16.2)	6 (20.7)	0 (0)	29 (16.6)	0.55
Symptomatology at presentation (*n* [%])	Cough	140 (98.6)	29 (100)	4 (100)	173 (98.9)	0.79
	Fever	101 (71.1)	19 (65.5)	4 (100)	124 (70.9)	0.36
	Night sweats	72 (50.7)	13 (44.8)	3 (75)	88 (50.3)	0.51
	Weight loss	48 (33.8)	14 (48.3)	1 (25)	63 (36)	0.3
	Anorexia	67 (47.2)	14 (48.3)	3 (75)	84 (48)	0.55
	Chest pain	46 (32.4)	10 (34.5)	1 (25)	57 (32.6)	0.93

*IQR* interquartile range, *n* number, *Rx* treatment, *SD* standard deviation, *TB* tuberculosis, *XP* Xpert MTB/RIF

*On 124, 26, 2 and 152 patients, respectively

There was a slight decrease in the total number of patients who would have been treated empirically (from 79 [20%] pre-XP to 72 [18%] post-XP). However, these were not the same individuals: 11 [38%] patients with a pre-XP empirical diagnosis were treated compared to 28 [20%] patients without a pre-XP diagnosis (Fig. 1). Patients with a pre-XP empirical diagnosis were more likely to be treated for TB despite a negative XP (post-XP empirical treatment, 7 [30%] versus 14 [11%], *P* = 0.014), however this effect disappeared on adjusting to the CD4 count (adjusted odds ratio [aOR] 2.5 [95% confidence interval, 0.8–8.0]; aOR per 50 cells/ul higher CD4 count 0.9 [95% CI 0.8–1.1]). There were no differences in baseline characteristics between those who were and were not treated empirically after a negative XP (Table 3), and no risk factors of empirical treatment could be identified in multivariable logistic regression (data not shown).

**Table 3:** Baseline characteristics of included smear-negative presumptive TB patients who had a negative Xpert® MTB/RIF result by actual empirical TB treatment initiation

Characteristic		Post-XP empirical Rx	No post-XP empirical Rx	Total	*P*-value
Total (*n* [%])		22	130	152	
Age (mean [SD])		36 (12)	41 (11)	40 (10)	0.04
Female sex (*n* [%])		13 (59.1)	71 (54.6)	84 (55.3)	0.70
CD4 count (median [IQR])*		239 (139, 329)	289 (133, 456)	283 (133, 441)	0.25
ART at presentation		12 (54.5)	69 (53.1)	81 (53.3)	0.90
Prior TB treatment (*n* [%])		1 (4.5)	25 (19.2)	26 (17.1)	0.09
Symptomatology at presentation (*n* [%])	Cough	21 (95.5)	129 (99.2)	150 (98.7)	0.15
	Fever	16 (72.7)	91 (70)	107 (70.4)	0.80
	Night sweats	11 (50)	61 (46.9)	72 (47.4)	0.79
	Weight loss	10 (45.5)	38 (29.2)	48 (31.6)	0.13
	Anorexia	13 (59.1)	55 (42.3)	68 (44.7)	0.14
	Chest pain	8 (36.4)	42 (32.3)	50 (32.9)	0.70

*IQR* interquartile range, *n* number, *Rx* treatment, *SD* standard deviation, *TB* tuberculosis, *XP* Xpert® MTB/RIF

*On 17, 118 and 135 patients, respectively

**Fig. 2:**
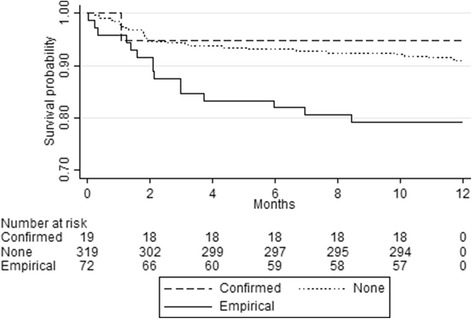
All-cause mortality in the first 12 months after presentation to the TB clinic among presumptive smear-negative TB patients, stratified by TB treatment on the basis of microbiological confirmation (positive XP), post-XP empirical treatment (negative or no XP) or no treatment (negative or no XP). Log-rank test for equality of survivor functions: *P* 0.01. Confirmed, microbiologically confirmed treatment; Empirical, post-Xpert® MTB/RIF empirical treatment; None, no treatment

Whilst simple analysis of mortality in the first year after presentation at the TB clinic was higher among those treated empirically (with no or negative XP result) compared to those treated with a positive XP or those not treated at all (22% versus 5% and 9% respectively, Fig. 2) there are multiple possible confounders of this relationship. In multivariable Cox regression analysis, adjusting for possible confounders that we had data for, including CD4 count, sex and ART usage at presentation, no association between post-XP empirical treatment or microbiologically confirmed treatment and the hazard of death was found (aHR 1.8 [95% CI, 0.8–3.9], and aHR 0.8 [95% CI, 0.1–6.3] respectively). For every 50 cells’ increase in CD4 count, it decreased by 18% (aHR 0.8 [95% CI, 0.7–0.9], *P* < 0.001). Male sex was associated with higher mortality (aHR 2.7 [95% CI, 1.3–5.9]), but no association was found for ART usage at presentation (aHR 0.5 [95% CI, 0.3–1.1]).

## Discussion

This study investigated the impact of XP usage on the diagnosis, treatment and mortality of HIV- positive smear-negative presumptive TB patients. Despite current WHO guidelines recommending the use of XP as the first diagnostic test rather than as an add-on to a negative sputum smear [3], this study offers insights into clinicians’ diagnostic and treatment decisions with the availability of XP and the associated treatment outcomes. We found that XP was only used in 43% of eligible patients despite free access to the test. The higher likelihood of an X-ray being ordered in this same group of patients suggests clinicians had a higher level of clinical suspicion compared to those not sent for XP. High levels of empirical treatment prevailed although XP testing did change who was ultimately treated for TB. A pre-XP empirical diagnosis by an experienced clinician did not predict XP positivity.

Although XP availability led to little change in the total proportion of presumptive TB patients actually treated for TB, the use of XP led to treatment of those who would not have been treated pre-XP and vice versa. Despite the clinicians’ experience, XP positivity was not more likely among patients with than without a pre-XP empirical diagnosis. This corroborates previous work on the poor accuracy of empirical diagnoses (using sputum culture as gold standard) in this clinic and in Tanzania [16, 17]. Despite having access to all clinical data, we were not able to determine which clinical factors most influenced clinicians in their decision-making process pre-XP. Among hospitalized patients, a recent study in Kampala identified productive cough, fever and tachycardia as predictors of empirical treatment [18]. The lack of sputum culture in our clinic precludes the determination whether empirical treatment was true- or false-positive (both pre- and post-XP).

Our data suggests that clinicians used the XP test as a “rule-in” test and only partly as a “rule-out” test. This is justifiable as the test is known to have low sensitivity as an add-on test to a negative sputum smear (61% in a HIV-positive population) [19, 20]. Nevertheless, the clinicians decided against treatment in just over half of the patients whom they would have treated without access to an XP, which may indicate an overestimated confidence in a negative XP. This potential false sense of security has also been described during roll-out of routine XP as the first-line diagnostic in South Africa [21]. This is likely to be exaggerated in rural areas where more mid-level health care workers are present with more limited TB diagnostic training [22]. In future, this risk might be mitigated by use of the next generation Xpert cartridge, the Xpert MTB/RIF Ultra, which has been shown to have a higher sensitivity [23].

Low XP utilisation has also been described in a study evaluating its use in 18 health facilities throughout Uganda (21%) [24], as well as in studies from the Democratic Republic of Congo (37%) and Swaziland (51%) [25, 26]. It has been suggested that the varying degrees of utilisation may be associated with the level of training and support provided to health care workers [24]. It may also take time for clinicians to get used to the new test. This has been shown in Swaziland where the utilisation increased to 73% in the second and third years after implementation, and in Cape Town, where the impact on empirical treatment reached its maximum in the third year after implementation [26, 27].

The lack of association between post-XP empirical treatment or microbiologically confirmed treatment and the risk of death was also found by three recent studies among both ambulant and hospitalized ART naïve patients [18, 28–30]. However, a recent systematic review on the impact of tuberculosis nucleic amplification tests, such as Xpert® MTB/RIF, concluded that the evidence has been of limited scientific rigour and from a relatively small number of settings, limiting its validity and generalisability [13]. We found that CD4 count confounded the negative association between empirical treatment and mortality. It is well known that a low CD4 count is a strong risk factor for mortality, and is likely to be associated with being treated empirically for TB [12]. Our study design was also not optimal to investigate this association, however, and we may not have captured potential confounding factors of the relationship between empirical treatment and mortality, including anemia, co-morbidities such as malignancies, and others.

The main limitation of this study was the risk of misclassification bias if clinicians failed to answer the clinical decision question truthfully due to misunderstanding of its purpose (whether it might affect the procedure in the laboratory or be used to review their performance). This was minimized with continued training and regular checks of their understanding. More than half of eligible patients were not sent for an XP; their higher probability of an EPTB diagnosis suggests a higher clinical suspicion of extrapulmonary involvement on the basis of additional information not captured in our data.

## Conclusions

XP usage to aid the diagnosis of smear-negative TB by experienced clinicians was lower than expected. In those tested, experienced clinicians mainly used the XP as a “rule-in” test and only partly as a “rule-out” test. The lower sensitivity of XP in smear-negative HIV-positive patients led to the majority of patients still being treated empirically.

## Abbreviations

aHR: adjusted hazard ratio
aOR: adjusted odds ratio
CD4: CD4+ T cell
HIV: Human Immunodeficiency Virus
IQR: Interquartile range
MDR: Multi-drug resistant
N: Number
Rx: Treatment
SD: Standard deviation
TB: Tuberculosis
WHO: World Health Organization
XP: Xpert MTB/RIF
